# Kv1.5 channels are regulated by PKC-mediated endocytic degradation

**DOI:** 10.1016/j.jbc.2021.100514

**Published:** 2021-03-04

**Authors:** Yuan Du, Tingzhong Wang, Jun Guo, Wentao Li, Tonghua Yang, Mark Szendrey, Shetuan Zhang

**Affiliations:** 1 Department of Cardiovascular Medicine, the First Affiliated Hospital of Xi'an Jiaotong University, Xi'an, China; 2 Department of Biomedical and Molecular Sciences, Queen's University, Kingston, Ontario, Canada

**Keywords:** Kv1.5, electrophysiology, endocytosis, ion channel, molecular biology, patch clamp, protein kinase C, structure–function relationships, ubiquitination, voltage-gated potassium channel, AF, atrial fibrillation, Baf, bafilomycin A1, BIM-1, bisindolylmaleimide I, CHMP3, charged multivesicular body protein 3, CTL, control, ESCRT, endosomal sorting complexes required for transport, HEK, human embryonic kidney, I_Kur_, ultra-rapidly activating delayed rectifier K^+^ current, I_Kv1.5_, Kv1.5 current, iPSC, induced pluripotent stem cell, Kv, voltage-gated potassium, Kv1.5-HEK, human embryonic kidney cells stably expressing Kv1.5 channel, MEM, minimum essential medium, MVB, multivesicular body, PBS, phosphate buffer solution, PKC, protein kinase C, PMA, phorbol 12-myristate 13-acetate, TBST, Tween-20 in Tris-buffered saline, Ub, ubiquitin, UbKO, lysineless Ub mutant, Vps24, vacuolar protein-sorting-associated protein 24

## Abstract

The voltage-gated potassium channel Kv1.5 plays important roles in the repolarization of atrial action potentials and regulation of the vascular tone. While the modulation of Kv1.5 function has been well studied, less is known about how the protein levels of Kv1.5 on the cell membrane are regulated. Here, through electrophysiological and biochemical analyses of Kv1.5 channels heterologously expressed in HEK293 cells and neonatal rat ventricular myocytes, as well as native Kv1.5 in human induced pluripotent stem cell (iPSC)-derived atrial cardiomyocytes, we found that activation of protein kinase C (PKC) with phorbol 12-myristate 13-acetate (PMA, 10 nM) diminished Kv1.5 current (I_Kv1.5_) and protein levels of Kv1.5 in the plasma membrane. Mechanistically, PKC activation led to monoubiquitination and degradation of the mature Kv1.5 proteins. Overexpression of Vps24, a protein that sorts transmembrane proteins into lysosomes *via* the multivesicular body (MVB) pathway, accelerated, whereas the lysosome inhibitor bafilomycin A1 completely prevented PKC-mediated Kv1.5 degradation. Kv1.5, but not Kv1.1, Kv1.2, Kv1.3, or Kv1.4, was uniquely sensitive to PMA treatment. Sequence alignments suggested that residues within the N terminus of Kv1.5 are essential for PKC-mediated Kv1.5 reduction. Using N-terminal truncation as well as site-directed mutagenesis, we identified that Thr15 is the target site for PKC that mediates endocytic degradation of Kv1.5 channels. These findings indicate that alteration of protein levels in the plasma membrane represents an important regulatory mechanism of Kv1.5 channel function under PKC activation conditions.

Voltage-gated potassium (Kv) channels play important roles in excitable cells and their malfunction can lead to various diseases. Kv1.5, encoded by *KCNA5*, is a member of the Shaker superfamily of Kv channels. Kv1.5 channels are expressed in a number of cell types such as atrial myocytes (1, 2), smooth muscle cells (3, 4), and pancreatic beta cells (5). In the heart, Kv1.5 channels conduct the ultra-rapidly activating delayed rectifier K^+^ current (I_Kur_), which is important for the repolarization of atrial action potentials (1, 2). Alterations in I_Kur_ are associated with atrial fibrillation (AF) (6, 7), and Kv1.5 channels are considered a promising target for pharmacological interventions to treat AF (8, 9).

Kv1.5 channels are regulated by various cellular molecules such as KChIP2 (10, 11) and posttranslational modifications such as phosphorylation (12) and SUMOylation (13). It has been shown that stimulation of α-adrenergic receptors can suppress I_Kur_ in human atrial myocytes *via* protein kinase C (PKC) activation (14). Since activation of PKC has been implicated in various cardiovascular diseases, such as cardiac hypertrophy and heart failure (15, 16), it is important to understand the mechanisms that underlie PKC-mediated regulation of Kv1.5 channels.

Previous studies have shown that PKC modifies Kv1.5 gating kinetics through phosphorylation of either auxiliary β subunits Kvβ1.2 (12, 17) or Kvβ1.3 (18). It has also been reported that PKC activation might decrease Kv1.5 surface expression though AMPK-Nedd4-2 pathway as well as an undisclosed mechanism (19).

In the present study, using phorbol 12-myristate 13-acetate (PMA) as a PKC activator, we investigated the effects of PKC activation on the expression and function of Kv1.5 channel expressed in human embryonic kidney (HEK) cells as well as neonatal rat ventricular myocytes. Our data revealed that PKC activation by PMA (10 nM) induces endocytic degradation of mature Kv1.5 channels through a lysosomal pathway. Using N-terminal truncation as well as site-directed mutagenesis, we identified that the threonine residue at position 15 in the N terminus is the target site for PKC-mediated endocytic degradation of Kv1.5 channels.

## Results

### PKC activation decreases Kv1.5 channel expression and Kv1.5 current (I_Kv1.5_)

**Figure 1:**
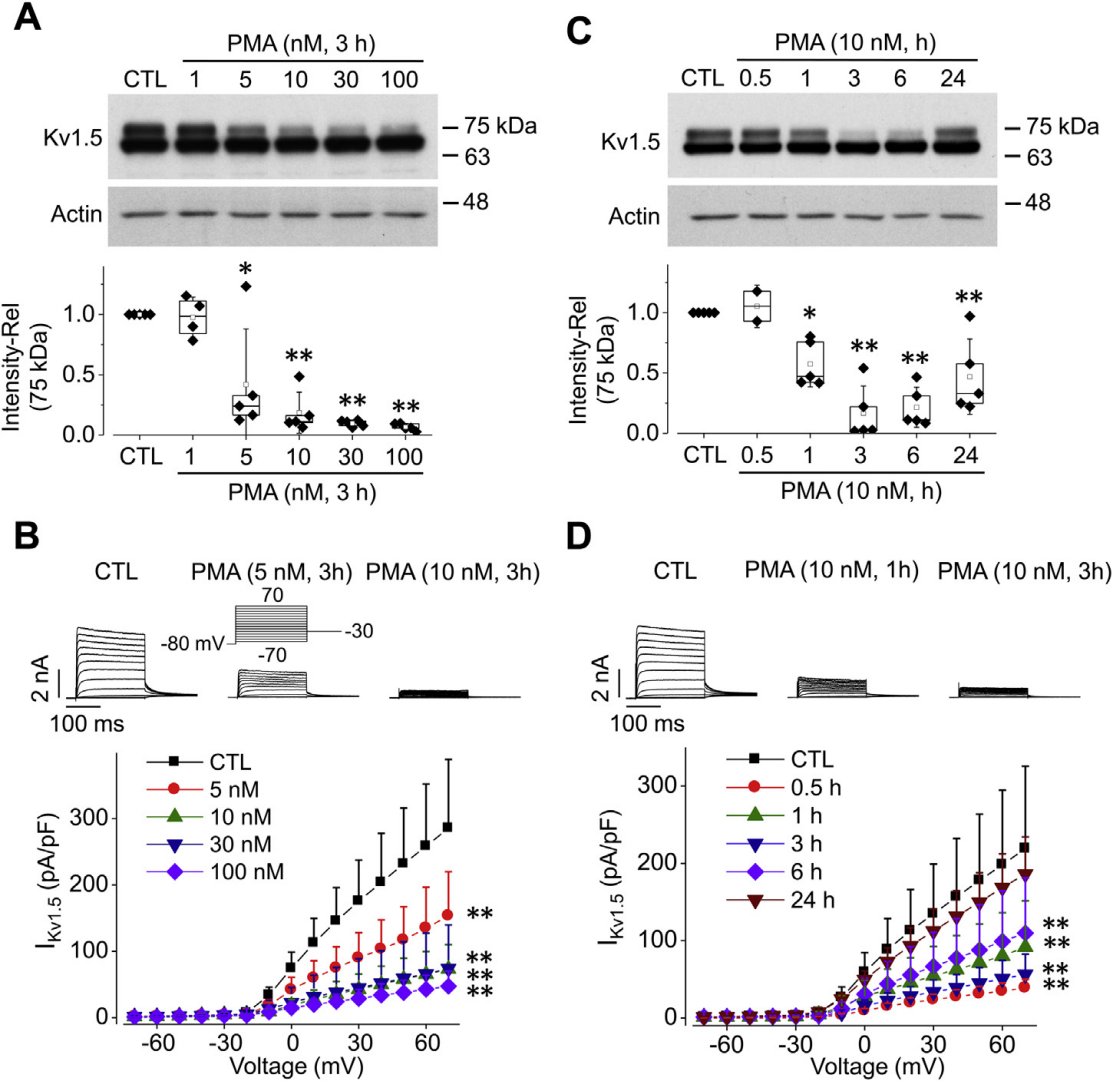
**PMA treatment decreases expression and current of Kv1.5 channels in HEK293 cells.***A* and *B*, concentration-dependent effects of PMA treatment for 3 h on the Kv1.5 expression (*A*, n = 5) and I_Kv1.5_ (*B*, n = 6–13 cells for each concentration). *C* and *D*, time-dependent effects of PMA (10 nM) on the Kv1.5 expression (*C*, n = 5) and I_Kv1.5_ (*D*, n = 7–14 cells for each time point). For western blot images, the actual molecular markers (the BLUeye Prestained Protein Ladder) are indicated beside the bands run on the same gels throughout the study. The 75-kDa band represents mature, fully glycosylated plasma membrane-located Kv1.5 channels, whereas the 68-kDa band represents core-glycosylated ER-located Kv1.5 channels. For western blot analysis, ∗*p* < 0.05, ∗∗*p* < 0.01 *versus* control (CTL). Rel, relative values. For current recordings, the voltage protocol shown in the inset of *B* is used throughout the study, pulse interval was 2 s ∗∗*p* < 0.01 *versus* CTL for current amplitudes upon >10 mV depolarizing steps. For western blots, data are presented as *box plots* and mean ± SD. For patch clamp, data are presented as mean + SD.

The phorbol ester PMA, a direct activator of PKC (12), was used to investigate the effects of PKC activation on Kv1.5 channels expressed in HEK293 cells. The cells were treated with PMA, and whole-cell proteins were analyzed using western blots. Kv1.5 protein displays two bands with molecular masses of ∼75 kDa and ∼68 kDa, due to the different glycosylation states (Fig. 1*A*, CTL). The 75-kDa band represents the mature fully glycosylated form localized in the plasma membrane, while the 68-kDa band is the immature core-glycosylated form of the Kv1.5 channel residing in the endoplasmic reticulum (ER). It is the plasma membrane localized, mature protein that conducts Kv1.5 current (I_Kv1.5_) (20). As shown in Figure 1*A*, treatment of cells with PMA at various concentrations for 3 h did not alter the expression level of the 68-kDa band, but decreased the 75-kDa band density in a concentration-dependent manner; 5 nM PMA significantly decreased Kv1.5 plasma membrane expression, 10 nM PMA caused a further decrease, but 30 nM or 100 nM PMA did not cause a further decrease.

To determine the effects of PMA treatment on Kv1.5 activity, we recorded I_Kv1.5_ using whole-cell patch clamp method. As shown in Figure 1*B*, consistent with the decrease in mature channel protein expression, PMA treatment for 3 h also reduced I_Kv1.5_ in a concentration-dependent manner.

To determine the time course of the PMA-induced reduction in Kv1.5 surface expression, cells were treated with 10 nM PMA and collected at different time points. PMA-induced mature Kv1.5 protein reduction became obvious at 1 h and reached a maximal effect at 3 h. Longer PMA treatment (6 h and 24 h) did not cause any further reduction, instead, mature Kv1.5 protein expression recovered partially at 6 h and almost fully at 24 h (Fig. 1*C*). The I_Kv1.5_ showed similar changes at various periods following PMA treatment (Fig. 1*D*). One possible explanation for these results is that while short periods of PMA treatment (*e.g.*, 3 h) activate PKC, long periods (*e.g.*, 24 h) lead to a depletion of certain PKC isoforms such as PKCα as we demonstrated recently (21), which allows Kv1.5 protein and function to recover.

### PKC inhibitors abolish PMA-induced reduction of Kv1.5 channels

**Figure 2:**
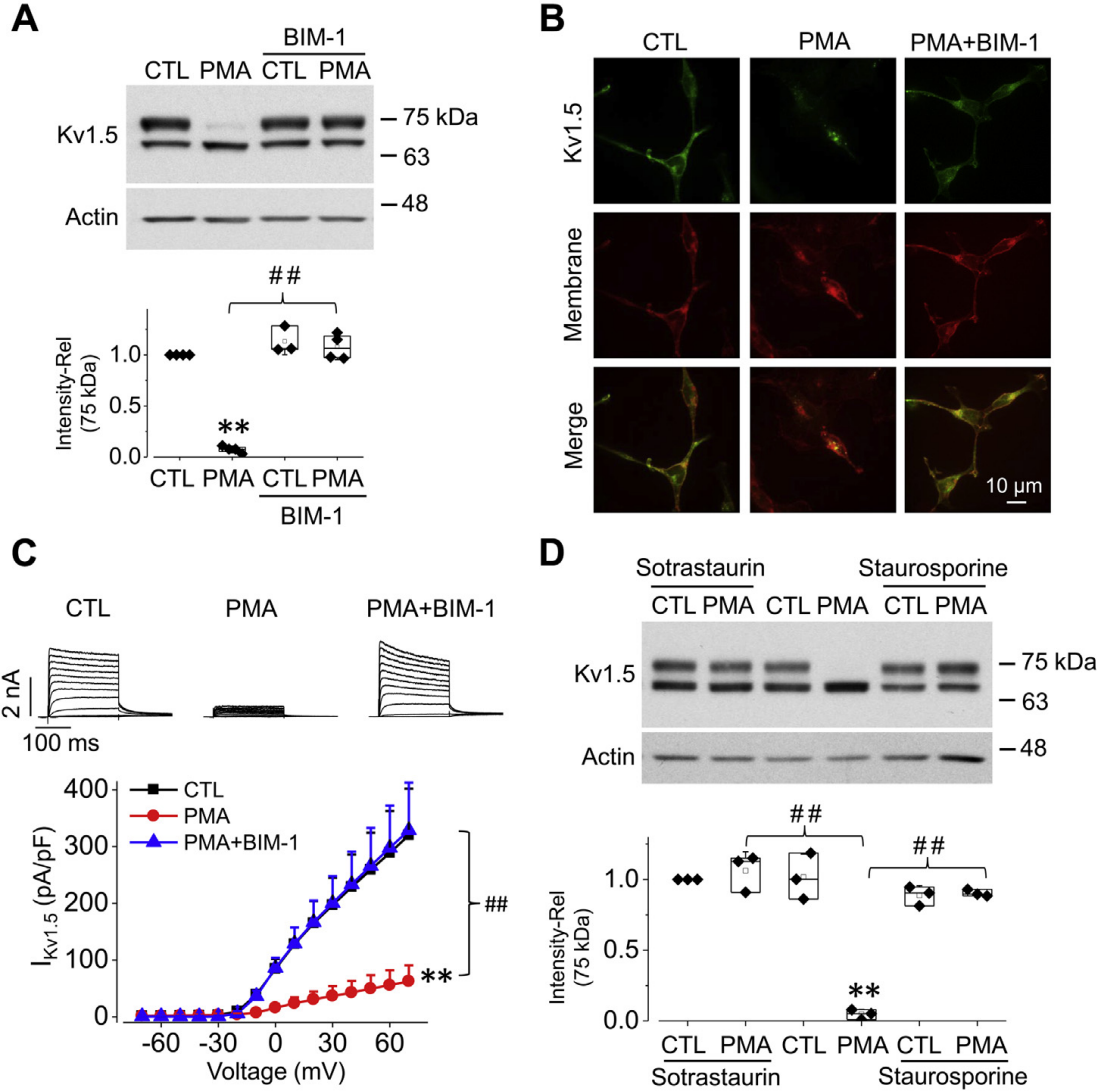
**PKC inhibitors abolish PMA-induced reduction in Kv1.5 expression and I**_**Kv1.5**_**in HEK293 cells.***A*, western blots result showing effects of 10 nM PMA treatment for 3 h on Kv1.5 expression with or without 10 μM BIM-1. Representative western blot images are displayed along with summarized data (n = 4). ∗∗*p*< 0.01 *versus* CTL; ^##^*p* < 0.01 *versus* PMA. *B*, confocal images showing effects of 10 nM PMA treatment for 3 h on Kv1.5 expression with or without 10 μM BIM-1. Kv1.5 was labeled with an anti-Kv1.5 primary antibody and Alexa Fluor 488-conjugated (*green*) secondary antibody. The cell membrane was labeled using a Texas Red X-conjugated WGA (*red*) (n = 4). *C*, effects of 10 nM PMA treatment for 3 h on I_Kv1.5_ with or without 10 μM BIM-1. Representative current traces are shown along with summarized I-V relationship. ∗∗*p* < 0.01 *versus* CTL; ^##^*p* < 0.01 *versus* PMA+BIM-1 for current amplitudes upon >10 mV depolarizing steps. n = 6–13 cells. *D*, western blots image showing the effects of 200 nM Sotrastaurin or 200 nM Staurosporine on Kv1.5 expression of Kv1.5-HEK cells treated with or without 10 nM PMA for 3 h (n = 3). ∗∗*p*< 0.01 *versus* CTL; ^##^*p* < 0.01 *versus* PMA. For western blots, data are presented as *box plots* and mean ± SD. For patch clamp, data are presented as mean + SD. Rel, relative values.

To validate that the PMA-induced effects on Kv1.5 are indeed though PKC activation, the PKC inhibitor, bisindolylmaleimide I (BIM-1), was used to prevent PKC activation (22, 23). While BIM-1 (10 μM) alone had no effect on Kv1.5 expression, it completely abolished the PMA-induced reduction in Kv1.5 mature channel expression (Fig. 2*A*).

To visualize the fate of Kv1.5 channels in the plasma membrane under PMA treatment, Kv1.5 was labeled with an anti-Kv1.5 primary antibody and Alexa Fluor 488-conjugated (green) secondary antibody. The cell membrane was labeled using Texas Red X-conjugated wheat germ agglutinin (WGA). As illustrated in Figure 2*B*, the *left lane*, in control cells, the majority of the Kv1.5 channels were present in the plasma membrane. In PMA-treated (10 nM, 3 h) cells, Kv1.5 channels displayed a diminished membrane expression and punctate intracellular localization (Fig. 2*B*, *middle lane*). In cells treated with both PMA (10 nM, 3 h) and BIM-1 (10 μM, 3 h), Kv1.5 channels remained in the plasma membrane (Fig. 2*B*, *right lane*).

BIM-1 also abolished PMA-induced I_Kv1.5_ reduction. The effects of PMA (10 nM, 3 h) on I_Kv1.5_ with or without BIM-1 (10 μM) were evaluated. As shown in Figure 2*C*, similar to the effect on Kv1.5 surface protein expression, BIM-1 (10 μM) abolished the PMA-induced I_Kv1.5_ reduction.

In addition to BIM-1, we also examined the effects of other PKC inhibitors Sotrastaurin and Staurosporine on PMA-induced reduction in Kv1.5 mature channel expression. Both Sotrastaurin (200 nM) and Staurosporine (200 nM) abolished the PMA-induced reduction of the mature Kv1.5 channel expression (Fig. 2*D*). Taken together, these findings indicate that the effects of PMA on Kv1.5 are mediated by PKC activation.

### Threonine at position 15 in the N terminus of Kv1.5 is essential for the effects of PKC activation on Kv1.5 channels

**Figure 3:**
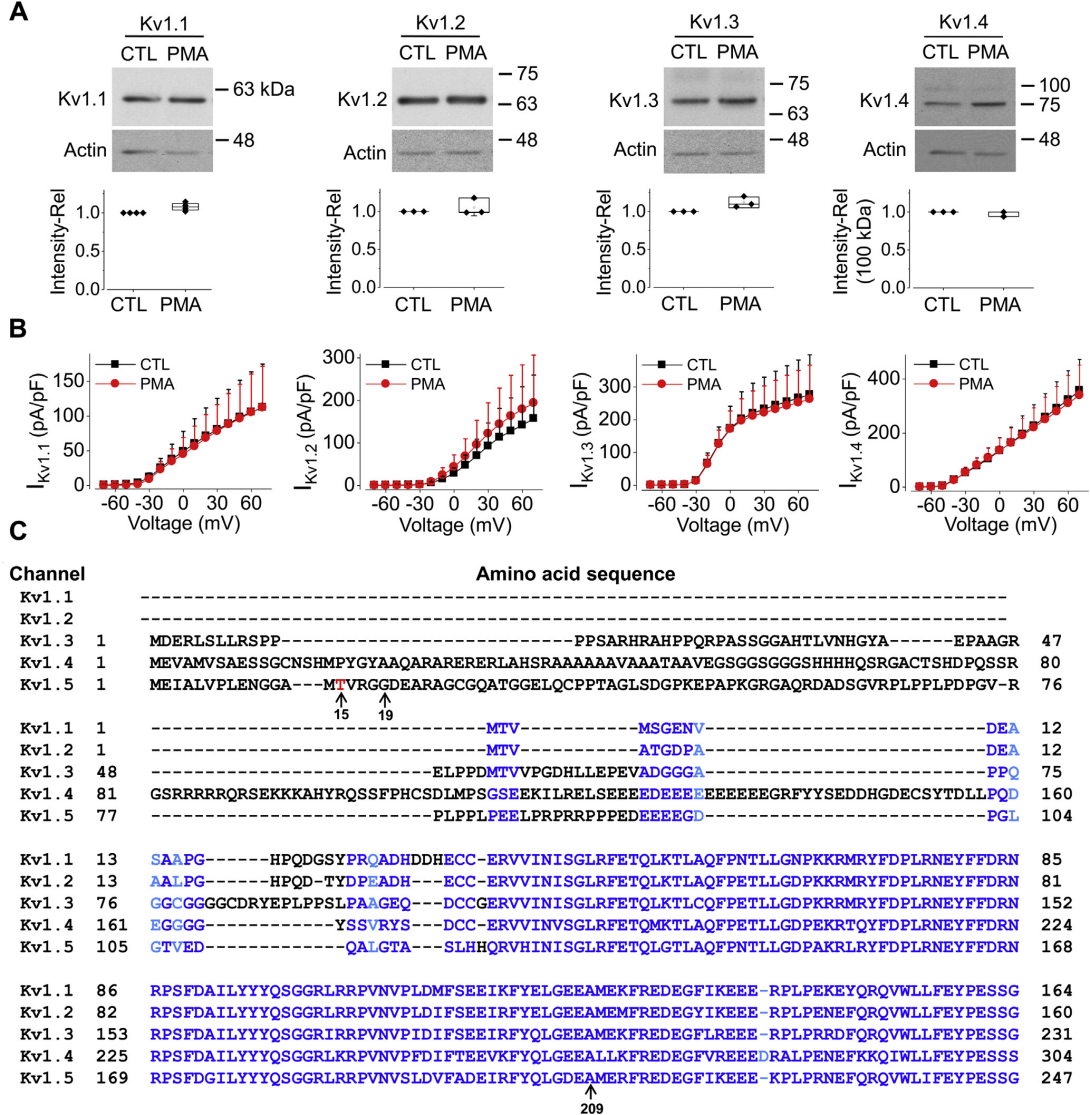
**PMA treatment (10 nM) for 3 h has no effect on Kv1.1, Kv1.2, Kv1.3, and Kv1.4 channels in HEK293 cells.***A*, western blot images showing the effect of PMA (10 nM) for 3 h on expressions of Kv1.1, Kv1.2, Kv1.3, and Kv1.4 channels stably expressed in HEK293 cells (n = 3–4). *B*, I-V relationships showing the effect of PMA treatment for 3 h on currents of Kv1.1, Kv1.2, Kv1.3, and Kv1.4 channels stably expressed in HEK293 cells (n = 7–18 cells for each channel type). For western blots, data are presented as *box plots* and mean ± SD. For patch clamp, data are presented as mean + SD. *C*, sequence alignment of the N terminus of Kv channels. Amino acid residues marked in blue are identical or similar among all channels; amino acid resides marked in *light blue* are different among channels; amino acid resides marked in black are segments that are absent in some channels. Threonine (T) at amino acid 15 in the N terminus of Kv1.5 is marked as *red*.

Kv1.5 belongs to Shaker superfamily of potassium channels. To test whether PKC activation also affects other Shaker superfamily members, we used PMA (10 nM) to treat HEK293 cells stably expressing Kv1.1, Kv1.2, Kv1.3, or Kv1.4 for 3 h. Our results showed that both protein expression and currents of these four channels were not affected by PMA treatments (Fig. 3, *A* and *B*). To determine the structural determinants involved in the unique PMA-induced Kv1.5 reduction, we first compared the amino acid residue differences among these Kv channels using COBALT: constraint-based alignment tool (NIH). A single Kv1.5 α-subunit contains six transmembrane domains (S1-S6), a cytoplasmic N-terminal and a C-terminal domain. We found that the greatest amount of differences is located in the N terminus among Kv channels (Fig. 3*C*), while the rest of the protein sequences among Kv channels are much more conserved. We hypothesized that residues within the N terminus in Kv1.5 channel are essential for PMA-induced reduction in Kv1.5 expression and current.

**Figure 4:**
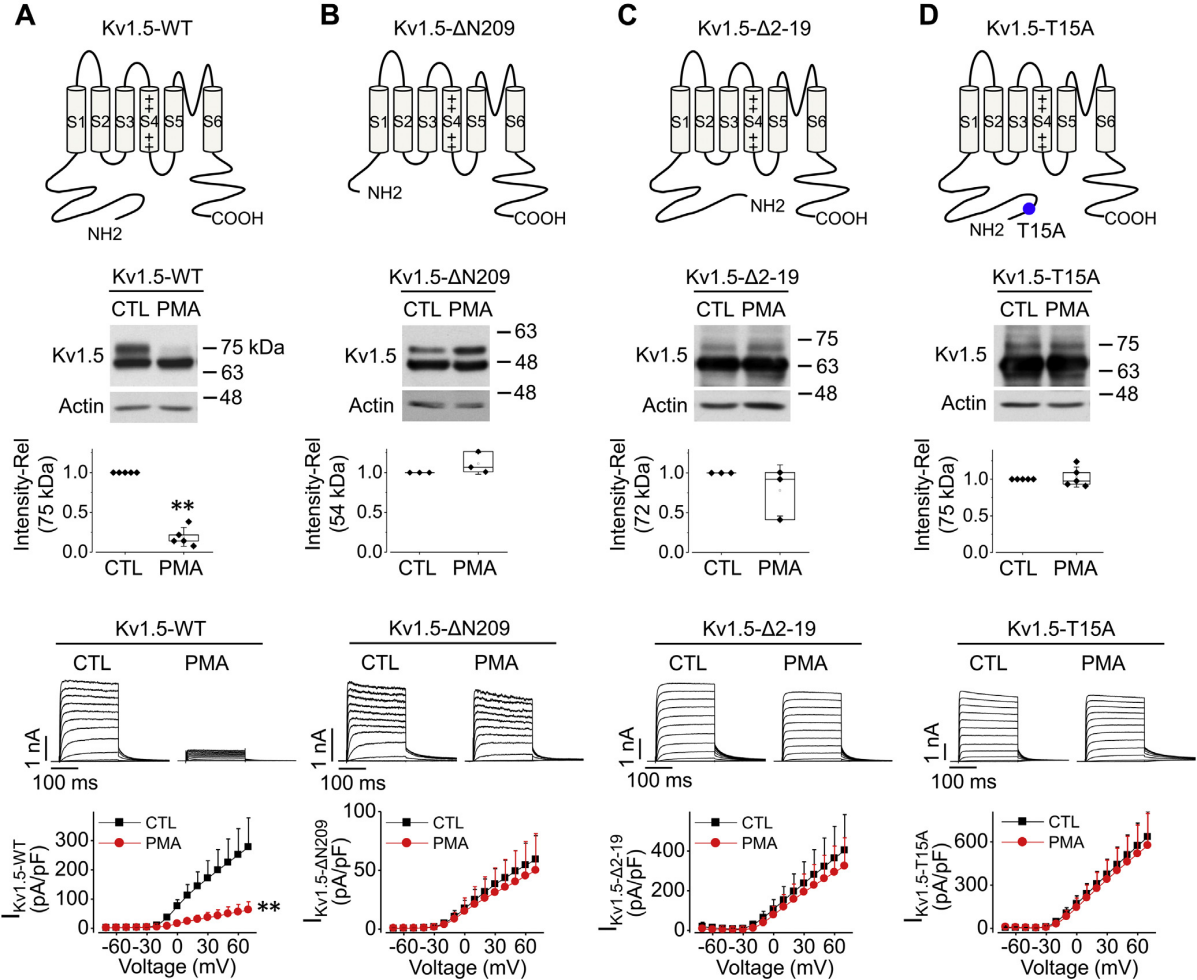
**Threonine at amino acid 15 in the N terminus of Kv1.5 is required for PMA-induced reduction in Kv1.5 expression and I**_**Kv1.5**_**in HEK293 cells.** For Kv1.5-WT (*A*), Kv1.5-ΔN209 (*B*), Kv1.5-Δ2-19 (*C*), and Kv1.5-T15A channels (*D*), schematic diagrams of channel structure are shown on the *top row*, western blot images are shown in the *middle row*, representative current traces along with summarized I-V relationships are shown in the *bottom row*. HEK293 cells stably expressing various channels were treated with 10 nM PMA for 3 h, and experiments were then performed. For western blots, n = 5 for WT, n = 3 for ΔN209, n = 3 for Δ2-19, n= 5 for T15A. ∗∗*p* < 0.01 *versus* CTL. For current recordings, n = 7–16 cells from three independent treatments for each channel. ∗∗*p* < 0.01 *versus* CTL for current amplitudes upon >10 mV depolarizing steps. For western blots, data are presented as *box plots* and mean ± SD. For patch clamp, data are presented as mean + SD.

To test this hypothesis, we truncated the N terminus of Kv1.5 up to residue 209 or deleted amino acid residues 2–19 to create Kv1.5-ΔN209 or Kv1.5-Δ2-19 mutants (Fig. 4, *A*–*C*). HEK293 cell lines stably expressing these respective truncation mutants were also established. We used PMA (10 nM) to treat Kv1.5-ΔN209-HEK cells and Kv1.5-Δ2-19-HEK cells, respectively, for 3 h and then performed western blots and patch clamp recordings. As shown in Figure 4, *A*–*C*, while PMA treatment decreased WT Kv1.5 expression and current, it affected neither expression nor function of these two mutant channels.

The fact that Δ2-19 deletion abolished the PMA-induced mature Kv1.5 reduction indicates that residues within the first 19 amino acid residues are involved in this process. PKC is involved in regulating the function of proteins through the phosphorylation of hydroxyl groups of the amino acids serine (S) and threonine (T) (24). There is only one threonine amino acid residue at position 15 (T15) within the first 19 amino acids of Kv1.5 channel. We thus made a point mutation by converting the threonine (T) to an alanine (A) to generate a Kv1.5-T15A mutant (Fig. 4*D*). When expressed in HEK293 cells, this mutation (Kv1.5-T15A), like the ΔN209 and Δ2-19, abolished the PMA-induced reduction of mature Kv1.5 expression and I_Kv1.5_ (Fig. 4). These results indicate that PMA activation targets the threonine residue at position 15 in the N terminus to induce the reduction of the mature Kv1.5 channel expression and I_Kv1.5_.

### PKC activation induces monoubiquitination of Kv1.5

**Figure 5:**
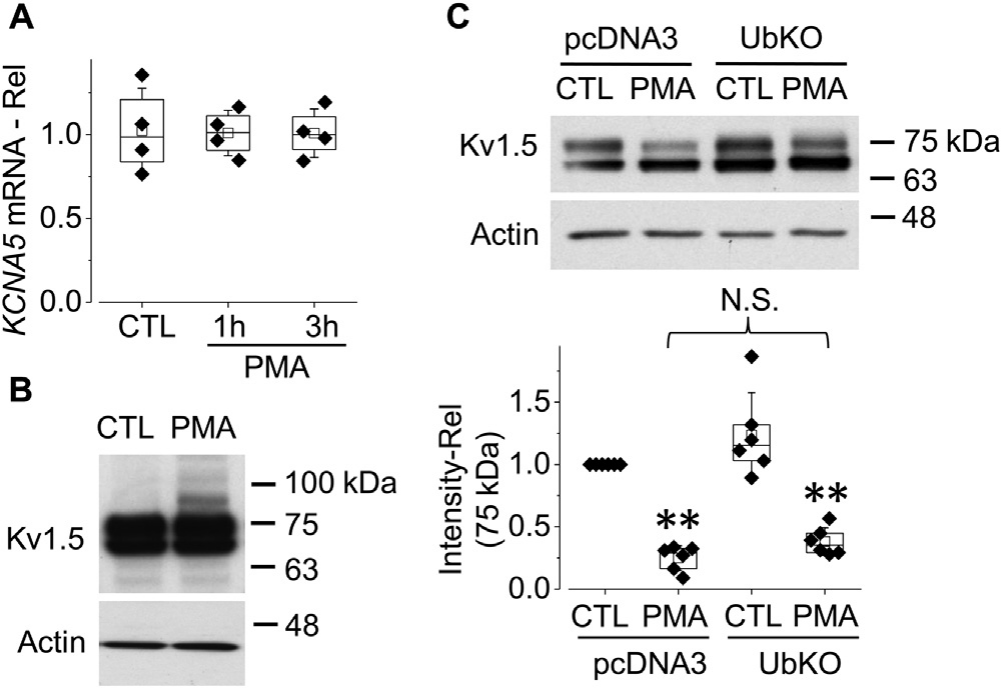
**PKC activation by PMA treatment induces monoubiquitination of Kv1.5 channels in HEK293 cells.***A*, effects of 10 nM PMA treatment on *KCNA5* mRNA levels detected using real-time PCR (n = 4). *B*, the western blot image showing that PMA treatment (10 nM, 0.5 h) induces an extra band, close to 83 kDa (n = 4). *C*, overexpression of UbKO has no effect on the PMA-induced reduction in expression of the 75-kDa form of Kv1.5 channels (n = 6). ∗∗*p* < 0.01 *versus* CTL. Data are presented as *box plots* and mean ± SD. N.S., not significant; Rel, relative values.

The homeostasis of Kv1.5 protein at the surface membrane is a balance between synthesis/anterograde trafficking and retrograde degradation. Mature Kv1.5 expression can be decreased through two mechanisms: reduced protein synthesis and/or accelerated Kv1.5 degradation. We examined the mRNA levels of *KCNA5* gene in control and PMA (10 nM, 1 h and 3 h) treated Kv1.5-HEK cells. As illustrated in Figure 5*A*, PMA did not decrease *KCNA5* mRNA level.

Ubiquitination mediates the degradation of various proteins including Kv1.5 channels (25, 26). In this process, ubiquitin (Ub) attaches to the target protein and triggers protein degradation. To examine whether Kv1.5 undergoes Ub modification upon PKC activation, we treated Kv1.5-HEK cells with PMA (10 nM) for a relatively short period (0.5 h) before notable reduction of mature Kv1.5 expression. As ubiquitination may occur continuously and concurrently with its mediated protein degradation, the fraction of ubiquitinated channels at a given time point may be small. We thus overexposed the membrane film to detect ubiquitinated Kv1.5 channel proteins. Western blot analysis of whole-cell Kv1.5 protein from PMA-treated cells displayed an additional band at 83-kDa (Fig. 5*B*). As the molecular mass of single Ub is 8.5-kDa, the 83-kDa band is most likely to be the monoubiquitinated membrane-localized 75-kDa Kv1.5. Monoubiquitination is known to trigger internalization and degradation of various channels (27, 28, 29).

In addition to monoubiquitination, in which a single Ub molecule is attached to the target protein, polyubiquitination (a chain of ubiquitin molecules binding to the target protein) also mediates channel protein degradation (30). To address the role of polyubiquitination in PMA-induced reduction of mature Kv1.5 expression, we transfected Kv1.5-HEK cells with a mutant Ub, UbKO, to disrupt polyubiquitination. In UbKO, all lysine residues are substituted to arginine, thus preventing the formation of polyubiquitin chains but allowing monoubiquitination (28, 29, 31). Twenty-four hours after transfection, the cells were exposed to 10 nM PMA for 3 h. As shown in Figure 5*C*, UbKO did not affect the PMA-induced reduction in expression of the mature form of Kv1.5 channels. These data indicate that it is monoubiquitination, not polyubiquitination, that mediates degradation of Kv1.5 channels upon PKC activation.

### Vps24 is involved in the PMA-induced Kv1.5 degradation

Monoubiquitinated proteins are sorted into the luminal vesicles of multivesicular bodies (MVBs) by endosomal sorting complexes required for transport (ESCRT) machinery (32). When MVBs fuse with the lysosome, the luminal contents are degraded (33). Vps24, also known as CHMP3 (charged multivesicular body protein 3), is a subunit of ESCRT-III in mammals that is essential for MVB formation and protein sorting (34).

**Figure 6:**
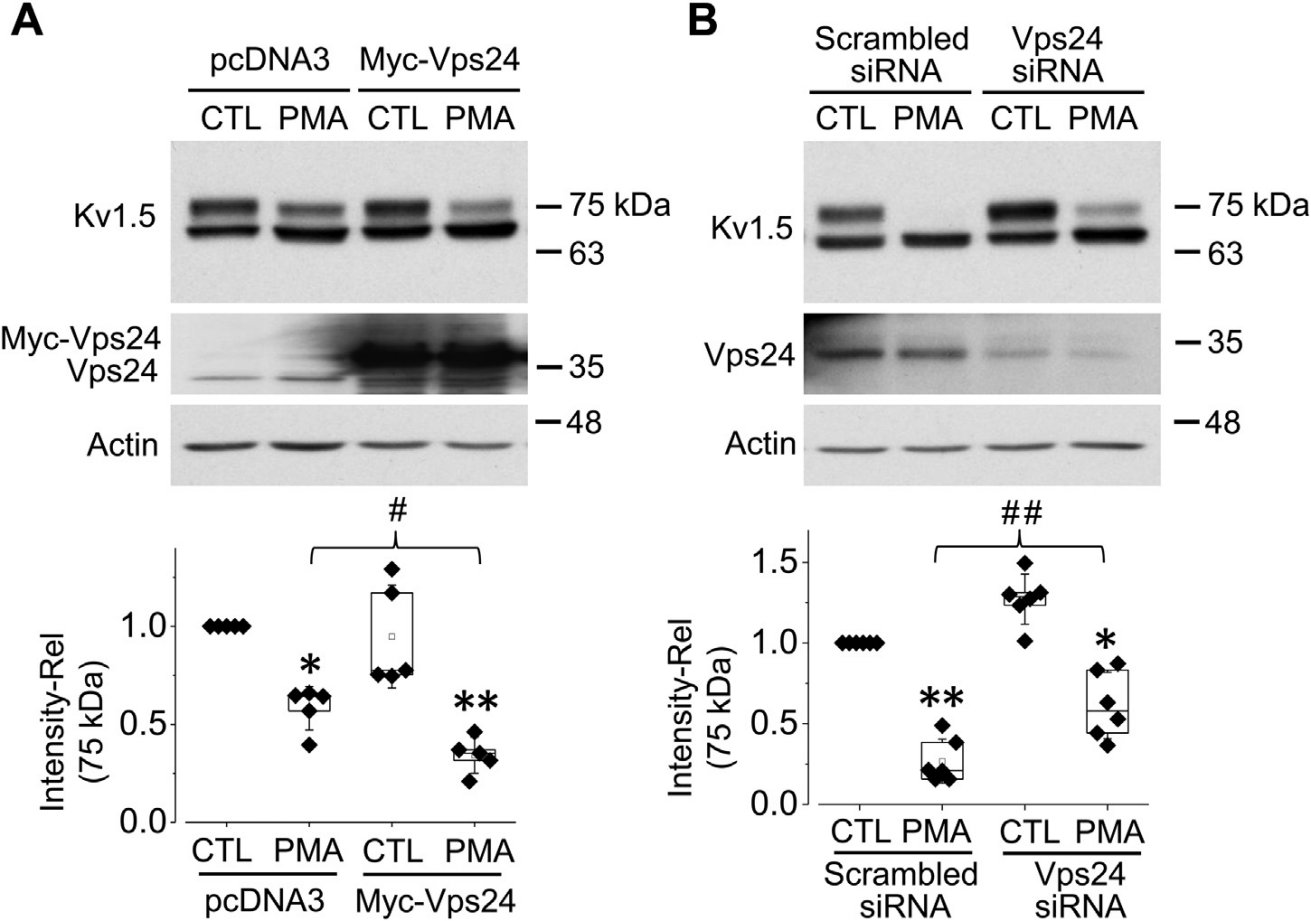
**Vps24 mediates PKC activation-induced Kv1.5 reduction in HEK293 cells.***A*, overexpression of Vps24 accelerates PMA (10 nM, 1 h)-induced Kv1.5 reduction. ∗*p* < 0.05, ∗∗*p* < 0.01 *versus* CTL (non-PMA treatment); ^#^*p* < 0.05 *versus* PMA in empty pcDNA3 transfected cells (n = 5). *B*, knockdown of endogenous Vps24 using siRNA slows down PMA (10 nM, 3 h)-induced Kv1.5 reduction. ∗*p* < 0.05, ∗∗*p* < 0.01 *versus* CTL (non-PMA treatment), ^##^*p* < 0.01 *versus* PMA in scrambled siRNA transfected cells (n = 6). Data are presented as *box plots* and mean ± SD. Rel, relative values.

We examined the effects of Vps24 overexpression on Kv1.5 degradation. Twenty-four hours after transfection of Kv1.5-HEK cells with Myc-tagged hVps24, cells were cultured in 10 nM PMA for 1 h, and Kv1.5 expression levels were examined. As shown in Figure 6*A*, overexpression of hVps24 enhanced PMA-induced reduction in the expression level of the 75-kDa Kv1.5 protein. To further examine the role of Vps24 in PMA-induced Kv1.5 reduction, we used Vps24 siRNA transfection to knock down the endogenous Vps24 protein expression. As shown in Figure 6*B*, Vps24 siRNA transfection notably reduced the endogenous Vps24 expression level and partially impeded the reduction of 75-kDa Kv1.5 expression induced by 10 nM PMA treatment for 3 h. Thus, Vps24 plays a role in the PMA-induced degradation of Kv1.5 channels.

### PMA-induced Kv1.5 degradation occurs through the lysosome

**Figure 7:**
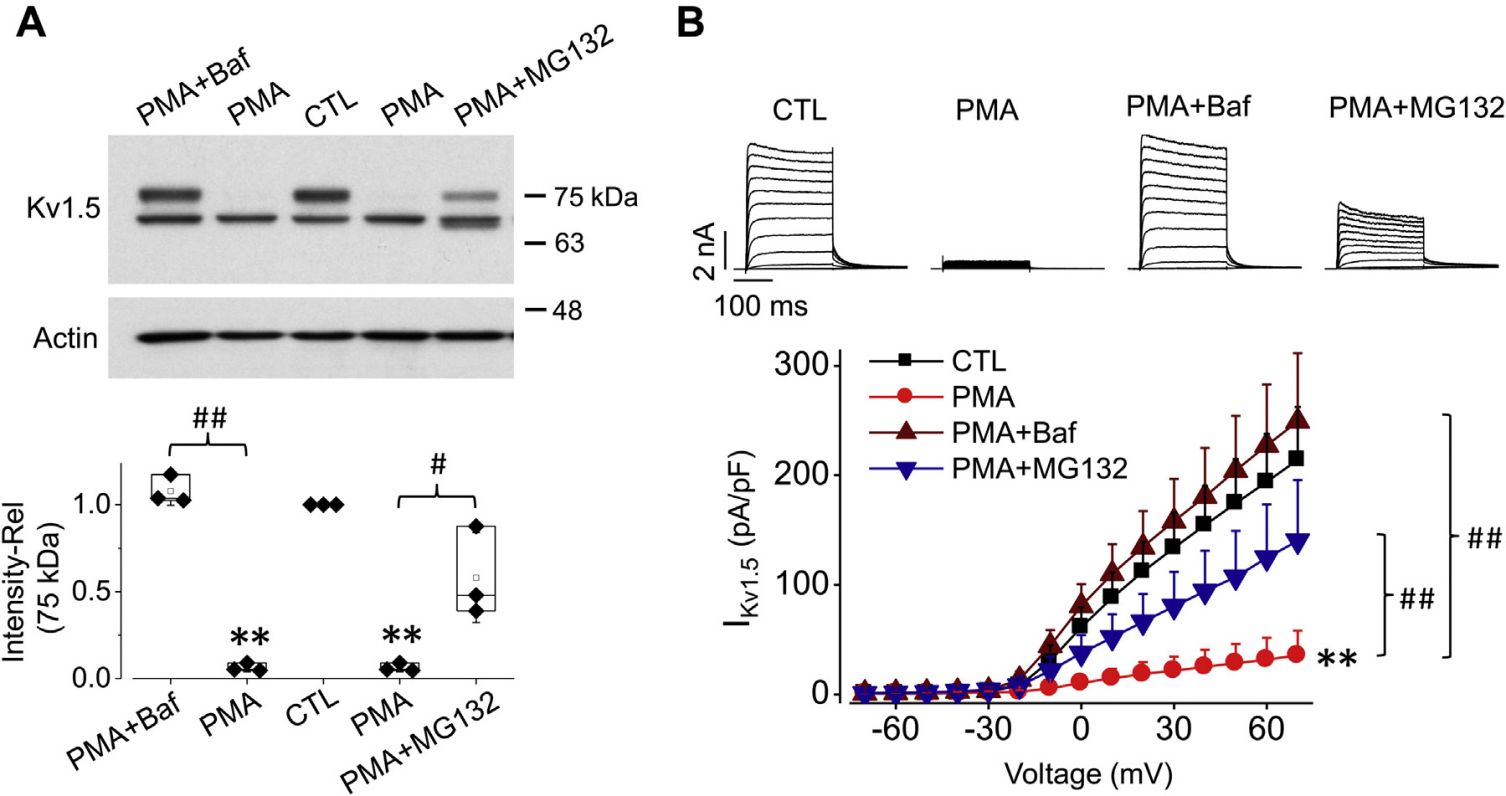
**Lysosomal inhibitor bafilomycin A1, but not proteasomal inhibitor MG132, completely abolishes PMA-induced reduction in Kv1.5 expression and I**_**Kv1.5**_**in HEK293 cells.***A*, effects of 10 nM PMA (3 h) on Kv1.5 expression with or without bafilomycin A1 (Baf, 1 μM) or MG132 (10 μM). Representative western blot images are displayed along with summarized data. n = 3. ∗∗*p*< 0.01 *versus* CTL; ^#^*p* < 0.05, ^##^*p*< 0.01 *versus* PMA. *B*, effects of 10 nM PMA (3 h) on I_Kv1.5_ with or without Baf (1 μM) or MG132 (10 μM). Representative current traces are displayed along with summarized I-V relationships (n = 7–26 cells). ∗∗*p* < 0.01 *versus* CTL; ^##^*p* < 0.01 *versus* PMA, for current amplitudes upon >0 mV depolarizing steps. For western blots, data are presented as *box plots* and mean ± SD. For patch clamp, data are presented as mean + SD. Rel, relative values.

Monoubiquitination is associated with the endocytosis and lysosomal sorting of plasma membrane proteins, while the formation of lysine 48 (K48)-linked polyubiquitin chains is the primary signal targeting proteins to the 26S proteasome for degradation (35). To test the notion that PMA-induced reduction of Kv1.5 is through lysosomal degradation, the lysosomal inhibitor, bafilomycin A1 (Baf, 1 μM), or the proteasomal inhibitor MG132 (10 μM) was included during PMA (10 nM) treatment of Kv1.5-HEK cells for 3 h. In western blot analysis, Baf completely abolished PMA-induced reduction in the expression of mature Kv1.5 channels, whereas MG132 only partially prevented the effect (Fig. 7*A*). Similar results were obtained in patch clamp recordings; while Baf completely prevented the PMA-induced I_Kv1.5_ reduction, MG132 only partially prevented PMA-induced I_Kv1.5_ reduction (Fig. 7*B*).

### PKC activation reduces currents of Kv1.5 channels transiently expressed in neonatal rat ventricular myocytes

**Figure 8:**
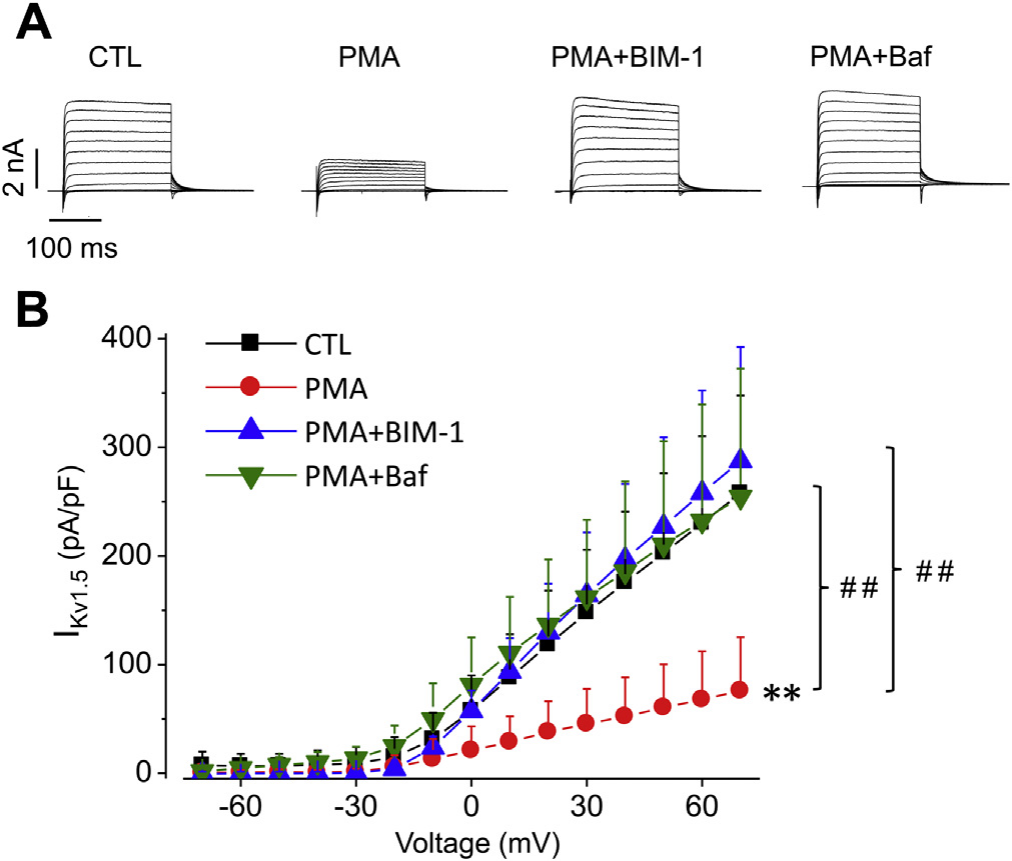
**PKC activation by PMA treatment decreases currents of Kv1.5 channels expressed in neonatal rat ventricular myocytes.** Representative current traces (*A*) and summarized current–voltage relationships (*B*) of I_Kv1.5_ recorded from Kv1.5-transfected neonatal rat ventricular myocytes treated with PMA (10 nM) for 3 h in the absence and presence of BIM-1 (10 μM) or Baf (1 μM) are shown. ∗∗*p* < 0.01 *versus* CTL; ^##^*p* < 0.01 *versus* PMA for current amplitudes upon >0 mV depolarizing steps. n = 9–12 cells in each group. Data are presented as mean + SD.

HEK293 cells have a different cellular environment from native cardiomyocytes. To determine whether PKC activation has a similar effect on Kv1.5 channels expressed in cardiomyocytes, we transfected Kv1.5 plasmid into isolated neonatal rat ventricular myocytes. Such transfection was necessary as Kv1.5 channels are primarily expressed in atria but not ventricles, the native I_Kur_ in neonatal rat ventricular myocytes is very small (36). In addition, it is difficult to isolate single atrial myocytes from neonatal rats due to the limited size of the tissues. I_Kv1.5_ recorded from transfected neonatal rat ventricular myocytes was robust and displayed similar biophysical properties to that from Kv1.5-HEK cells. As shown in Figure 8, PMA treatment (10 nM, 3 h) decreased I_Kv1.5_, while presence of PKC inhibitor BIM-1 (10 μM), or lysosomal inhibitor Baf (1 μM), prevented PMA-induced I_Kv1.5_ reduction. These results are consistent with those observed in Kv1.5-HEK cells. Thus, PKC activation decreases I_Kv1.5_ in both the heterologous expression system (HEK293 cells) and a more physiological environment (cardiomyocytes).

### PKC activation reduces I_Kv1.5_ in human induced pluripotent stem cell (iPSC)-derived atrial cardiomyocytes

**Figure 9:**
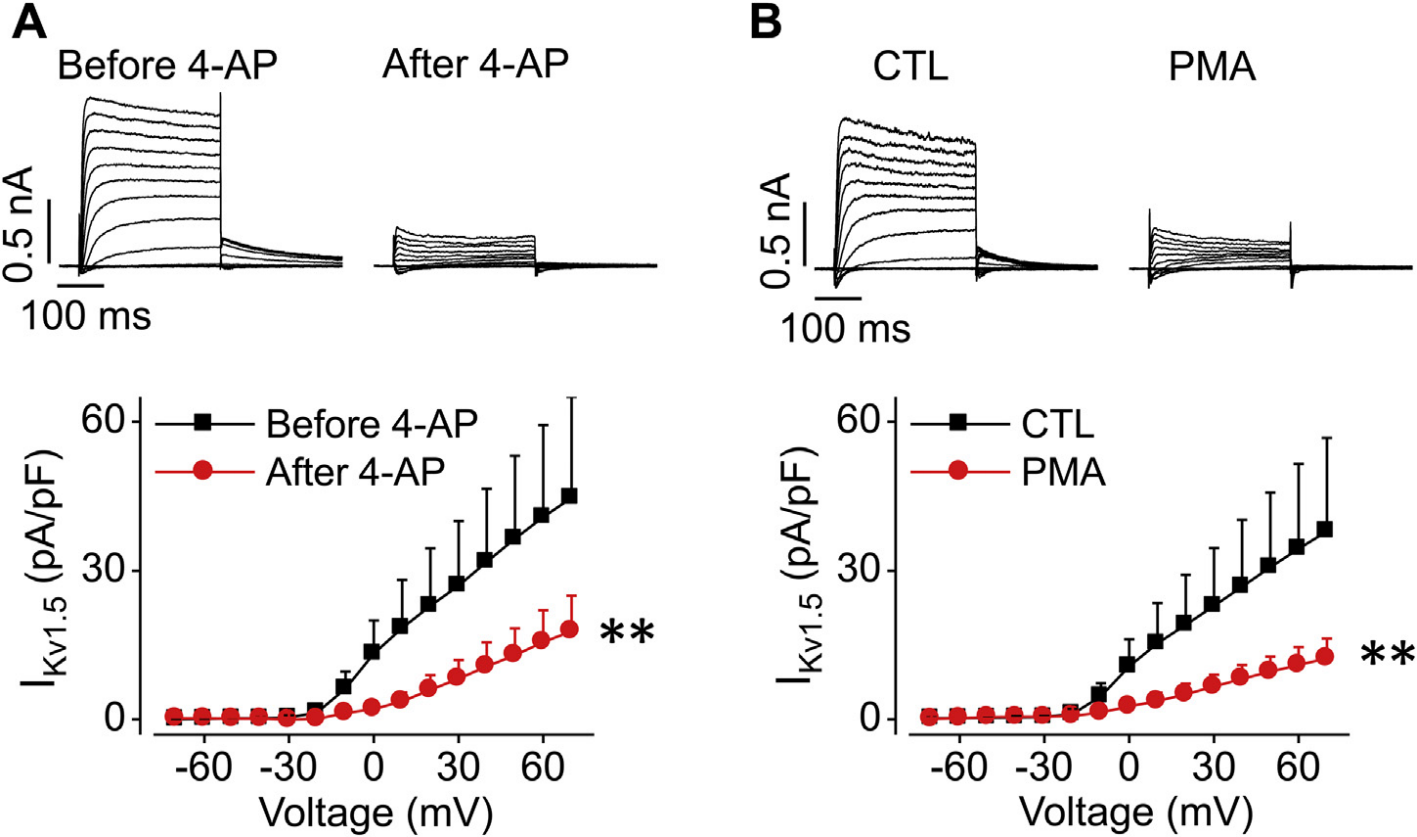
**PKC activation by PMA treatment decreases I**_**Kv1.5**_**in human iPSC-derived atrial cardiomyocytes.***A*, representative current traces and summarized current–voltage relationships of I_Kv1.5_ before and after 0.1 mM 4-AP application. After currents were recorded in control conditions (before 4-AP), 0.1 mM 4-AP was applied to the bath solution for 2 min to achieve stead-state block, and currents were recorded in the same cell in the presence of 4-AP (after 4-AP). n = 5. ∗∗*p* < 0.01 *versus* before 4-AP for current amplitudes upon >0 mV depolarizing steps. *B*, representative current traces and summarized current–voltage relationships of I_Kv1.5_ in control cells (n = 15 cells) and cells treated with 10 nM PMA for 3 h (n = 13 cells). After treatment, cells were transferred to the recording chamber superfused with the external solution. The voltage protocol is the same as shown in Figure 1*B*. ∗∗*p* < 0.01 *versus* CTL for current amplitudes upon >0 mV depolarizing steps. For *A*, a two-tailed paired Student's *t*-test was used. For *B*, a two-tailed unpaired Student's *t*-test was used. Data are presented as mean + SD.

To further confirm the effect of PKC activation on native I_Kv1.5_, human iPSC-derived atrial cardiomyocytes were used. Most cells during culture from day 3 to day 6 displayed the K^+^ current with biophysical and pharmacological characteristics of I_Kv1.5_. The activation–voltage relationships of I_Kv1.5_ revealed a half-maximal activation voltage (V_1/2_) of -13.2 ± 3.6 mV with a slope factor of 5.0 ± 1.2 (n = 12) in human iPSC-derived atrial cardiomyocytes and a V_1/2_ of -7.0 ± 2.5 mV with a slope factor of 5.6 ± 0.6 (n = 12) in Kv1.5-HEK cells. As well, the current displayed slow inactivation during 200-ms depolarizing steps between 30 and 70 mV. Furthermore, as shown in Figure 9*A*, the current was sensitive to the Kv1.5 channel blocker 4-aminopyridine (4-AP); 0.1 mM 4-AP blocked the current by more than a half (Fig. 9*A*); and the block was reversible (data not shown). We then paired control and PMA-treated (10 nM, 3 h) myocytes each time and performed whole-cell voltage clamp recordings. As shown in Figure 9*B*, treatment with PMA (10 nM, 3 h) reduced the I_Kv1.5_ by 67% (38.0 ± 18.8 pA/pF in CTL (n = 15), 12.3 ± 4.0 pA/pF in PMA (n = 13), *p* < 0.01). There are two possibilities regarding the remaining currents in PMA-treated myocytes: PMA at 10 nM did not completely inhibit I_Kv1.5_; or, a small amount of PMA-insensitive currents other than I_Kv1.5_ may contribute to currents in these cells.

## Discussion

Kv1.5 is important for atrial action potential repolarization, and its dysfunction caused by gene mutations has been linked to atrial fibrillation (37). In the present study, our results showed that PKC activation by PMA decreased I_Kv1.5_ channels expressed in HEK293 cells as well as in rat cardiomyocytes and native I_Kv1.5_ in human iPSC-derived atrial cardiomyocytes. Further detailed analyses in WT and various mutant Kv1.5 channels expressed in HEK293 cells revealed that PKC activation enhanced cell-surface Kv1.5 internalization and degradation through the ubiquitination/MVB/lysosome pathway, which thereby led to decrease of Kv1.5 current and surface protein expression. Additionally, the intact Threonine residue at position 15 (T15) in the N terminus of Kv1.5 is required for this process.

Previous studies have demonstrated that PKC activation could suppress Kv1.5 current through direct phosphorylation of the accessory β-subunits (12, 17, 18, 38). It was demonstrated that PMA had minimal effect when Kv1.5 was expressed alone. However, K^+^ currents derived from coexpression of Kvβ1.2 with Kv1.5 were markedly reduced by PMA, and the reduction was associated with a small depolarizing shift in the voltage dependence of channel activation (12). In our experiments, only the α subunit Kv1.5 was transfected into HEK293 cells, therefore PKC activation may directly act on Kv1.5 independently of the β subunits. Our data obtained using western blot (Fig. 2*A*) and confocal analysis (Fig. 2*B*) demonstrated that the PKC-induced decrease in Kv1.5 activity is due to downregulation of the cell surface Kv1.5 channels. These findings are consistent with a recently published report (19). In brief, in addition to the direct phosphorylating effect of PKC on the accessory β-subunits, a novel mechanism for Kv1.5 suppression was PKC activation-induced internalization of cell surface channels. While the former modulates Kv1.5 activity by changing the single-channel functional properties (qualitatively), the latter regulates the number of surface ion channels (quantitatively).

The effects of PKC activation on Kv1.5 channel are similar to that of quinidine, an antiarrhythmic drug. Quinidine directly inhibits ionic current of Kv1.5 channels by pore blocking. It also decreased the number of Kv1.5 channels on the cell surface through channel internalization (39). Besides Kv1.5 channels, the surface densities of other potassium channels are affected by PKC activation. For example, activation of PKC stimulates endocytosis of KCNQ1-KCNE1 complex (I_Ks_) (40) and K_ATP_ channel (41).

The internalized membrane proteins are either recycled back to the plasma membrane or subjected to degradation (42). There are two main cellular pathways for protein degradation: proteasomal and lysosomal degradation pathways. Polyubiquitinated proteins are targeted for degradation by the proteasome (43), whereas monoubiquitination is sufficient to trigger endocytic degradation of membrane proteins in lysosomes (44). Our data showed that PMA treatment induced monoubiquitination of Kv1.5 (Fig. 5*B*) and the reduction effect of PMA on Kv1.5 was not weakened by UbKO overexpression, which disrupts polyubiquitination of target proteins (Fig. 5*C*). These results indicate the monoubiquitination of the mature Kv1.5 channels under PKC activation conditions. Monoubiquitination acts as a sorting signal for internalization and lysosomal degradation of membrane proteins. Once protein cargo is tagged by a Ub molecule, it is recognized and sorted into luminal vesicles of MVBs, which then fuse with lysosome through the ESCRT sorting machinery (45, 46). Vps24 is an important member of ESCRT-III. In the present study, overexpression of Vps24 accelerated the PKC activation-induced 75-kD Kv1.5 degradation (Fig. 6*A*), whereas knockdown of endogenous Vps24 with siRNA transfection markedly impeded the degradation (Fig. 6*B*). These results suggest that the ESCRT component, Vps24, is involved in transport of the Kv1.5 channels from early endosomes to lysosomes for degradation under PKC activation conditions.

**Figure 10:**
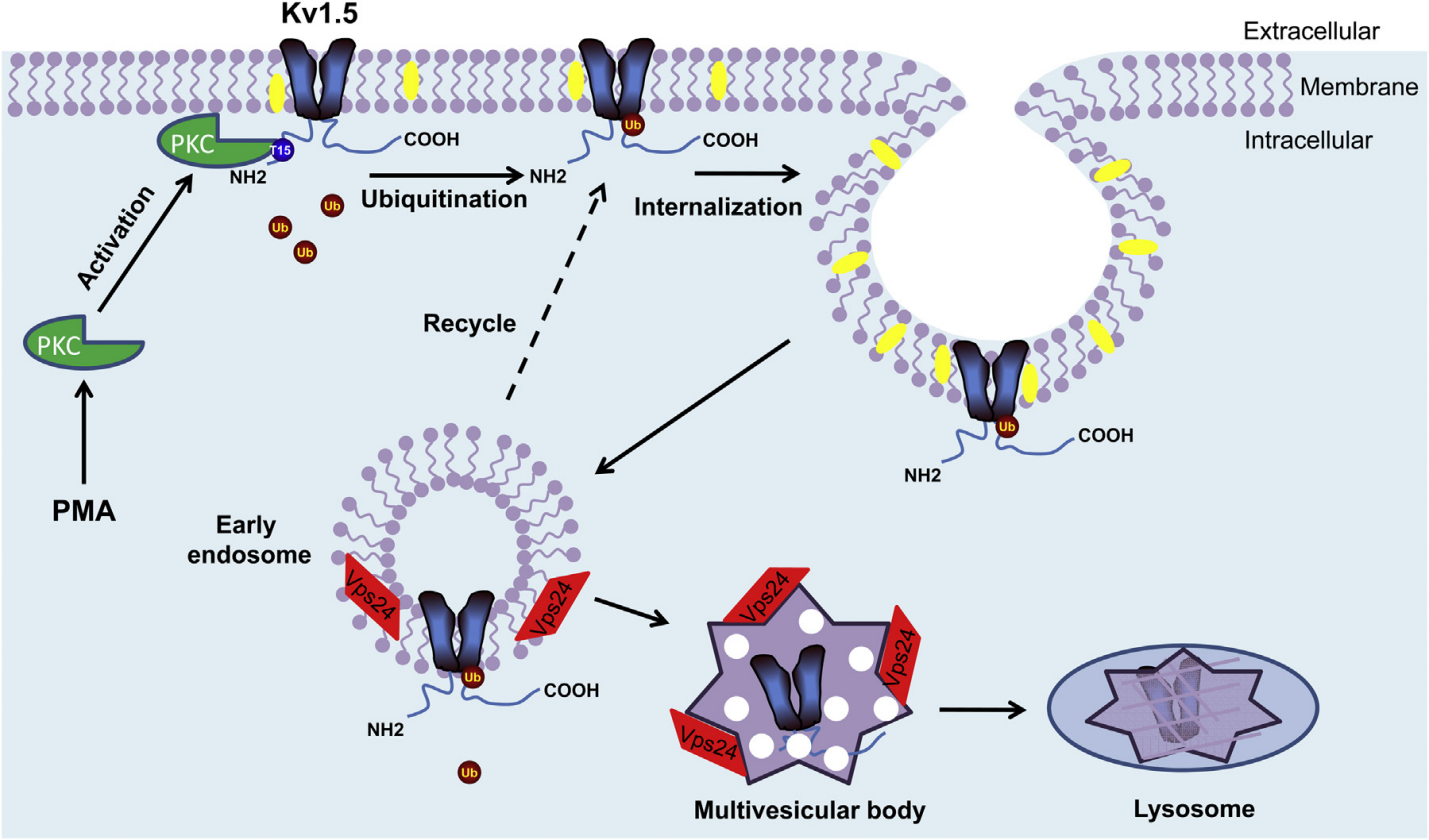
**Schematic illustrating the process of PKC activation-induced endocytic degradation of Kv1.5 channel.** PKC activation targets T15 at the N terminus of Kv1.5 channel on the surface membrane and induces monoubiquitination of the channel. Ubiquitinated channel undergoes internalization through MVB/Vps24 into lysosome to degradation.

Ultimately, Kv1.5 channels were degraded in the lysosome. This is supported by our data that lysosomal inhibitor (bafilomycin A1) completely prevented PMA-induced reduction in Kv1.5 expression and current (Fig. 7). Prevention of Kv1.5 degradation in lysosome would impede the endocytosis of cell-surface Kv1.5 channels through increasing endosomal recycling to the plasma membrane (Fig. 10). In addition, we also found that PKC activation-induced Kv1.5 internalization was partially impeded by the proteasomal inhibitor MG132 (Fig. 7). The reason for the partial preventive effects of MG132 is unknown. However, MG132 might inhibit some enzymes that are important for the sorting of ubiquitinated cargo proteins in MVBs (47). Impairment of protein sorting may lead to a larger pool of internalized mature proteins in the early endosome, which increases protein recycling to the cell surface (48). Overall, our results indicate that the monoubiquitination/MVB/lysosome pathway is responsible for Kv1.5 degradation when PKC is activated.

To identify the structural determinants involved in PKC activation-induced Kv1.5 reduction, we studied responses of other Kv1 channels to PKC activation. We found that Kv1.5, but not Kv1.1, Kv1.2, Kv1.3, or Kv1.4, was uniquely sensitive to PMA treatment (Fig. 3, *A* and *B*). Sequence alignment results suggested that residues within the N terminus in Kv1.5 channel are essential for PKC activation-induced Kv1.5 reduction (Fig. 3*C*). We then utilized mutant channel Kv1.5-ΔN209 in which amino acid residues up to 209 in the N terminus has been truncated (20). Since this truncation completely abolished PMA-induced Kv1.5 reduction (Fig. 4*B*), we further searched the target region for PKC activation. We focused on the proline-rich sequence existing at position 65–82 in Kv1.5 N terminus. Because Src tyrosine kinase targets this proline-rich sequence that is important for Kv1.5 endocytosis (49, 50), and PKC can indirectly activate Src (51), we tested a hypothesis that PKC activation reduces Kv1.5 protein *via* proline-rich sequence. We thus disrupted the proline-rich sequence by making P68/69A and P79/80A mutants as well as Kv1.5-ΔPro: deletion of amino acids 64 to 82 (49, 52). However, our results showed that while disrupting the proline-rich sequence abolished Src-induced effects on Kv1.5 channel, it had no effect on PMA-induced reduction of the channel (data not shown). Thus, proline-rich sequence did not seem to be involved in the PKC activation-mediated internalization and degradation of Kv1.5 protein.

To further narrow down the site for PKC activation-induced reduction in Kv1.5 density, we focused on the potential PKC consensus phosphorylation motif (S/T-X-R/K) TVR (residues 15–17) in the N terminus. Previous studies showed that SAP97 (synapse-associated protein 97) increases I_Kv1.5_, and this effect can be blocked either by disrupting the potential PKC consensus phosphorylation motif through T15A mutation or by pretreatment of cells with a PKC inhibitor (53, 54). In the present study, we demonstrate that T15 residue at N terminus is necessary for PKC-induced Kv1.5 internalization (Fig. 4*D*). It has been demonstrated that phosphorylation of the norepinephrine transporter (NET) at T258 and S259 is linked to PKC-mediated transporter internalization (24). Furthermore, ubiquitination has been found to be critical for the PKC-dependent endocytosis of some transporter proteins, such as dopamine transporter (DAT) (55) and glutamate transporter-1 (GLT-1) (56).

Our results showed that acute application of PMA (10–100 nM) to cells during whole-cell clamp recordings for up to 30 min did not cause a reduction in current (data not shown). This indicates that an intact cellular environment is required for PKC-mediated modulation of the channel. We believe that T15 residue serves as a PKC phosphorylation site. It would be interesting to investigate how the phosphorylation of T15 residue by PKC activation causes the subsequent Kv1.5 channel ubiquitination and endocytosis. In this regard, our results showed that PMA-treatment-induced reduction in I_Kv1.5_ occurred earlier than the reduction of mature protein (Fig. 1, *C* and *D*). At least two possibilities exist: (1) PMA induces a loss of Kv1.5 function, which triggers protein internalization; (2) PMA induces Kv1.5 protein internalization, which results in a loss of Kv1.5 function, and internalized channels then degrade at rate slower than internalization. Further study will be needed to distinguish these possibilities. As well, T15A mutant displays a higher current density than WT Kv1.5 probably due to the disruption of the basal level of PKC-mediated degradation. However, although it would be expected this mutant to have higher surface expression, our western blots show that the apparent ratio of mature to immature channels is lower than wild-type (WT) channel (Fig. 4). The exact reason for this phenomenon is unknown. It may be due to the fact that the total expression of T15A mutant is much higher than WT; or T15A mutation may behave differently not just in terms of trafficking. Further investigation may be warranted in future studies.

PKC activation occurs in various physiological and pathophysiological conditions (16). For instance, the levels of PKCα and PKCβ isoforms are elevated during heart failure (57) while upregulation of PKC isoforms α and ε is observed in cardiac hypertrophy (58). In addition, high protein level of PKCε was detected in chronic atrial fibrillation (59). Our finding that PKC activity controls Kv1.5 density in the plasma membrane provides a potential mechanistic link between various cardiovascular pathologies and high prevalence of atrial arrhythmias.

In summary, we have demonstrated that PKC activation leads to a reduction in cell surface expression of the voltage-gated potassium channel Kv1.5 *via* endocytic degradation. This process relies on the presence of T15 residue at the Kv1.5 N terminus. To the best of our knowledge, this is the first study providing detailed insight into the intracellular pathways that regulate Kv1.5 channels *via* PKC activation, and this finding extends our understanding of ion channel biology and provides a potential mechanism for the development of atrial arrhythmias in multiple cardiac pathologies.

## Experimental procedures

### Molecular biology

WT Kv1.5 cDNA was provided by Dr Michael Tamkun (Colorado State University, Fort Collins, CO). For generating plasmid Δ2-19 (deletion of 2–19), ΔN209 (truncation of 1–209), and T15A, PCR cloning of the corresponding inserts from WT Kv1.5 template was constructed into pcDNA3 using BamH1 and EcoR1 restriction enzymes. The constructs were verified through sequencing across the inserts (GENEWIZ). The human lysineless mutant Ub (UbKO; plasmid number 17603) was obtained from Addgene. Human Vps24 cDNA open reading frame (ORF) clone with Myc tag in pCMV6-entry vector (Myc-Vps24; plasmid number RC220006) was obtained from OriGene Technologies. FLAG-tagged Kv1.1, Kv1.2, Kv1.3, and Kv1.4 cDNAs were purchased from GenScript.

Lipofectamine 2000 was used to transfect WT Kv1.5 and mutant constructs with GFP plasmid into human embryonic kidney (HEK) 293 cells. Cell lines stably expressing Kv1.5-WT (Kv1.5-HEK), Kv1.5-ΔN209 (Kv1.5-ΔN209-HEK), Kv1.5-Δ2-19 (Kv1.5-Δ2-19-HEK), and Kv1.5-T15A (Kv1.5-T15A-HEK) were established using G418 for selection (1 mg/ml) and maintenance (0.4 mg/ml). HEK cells were cultured in Minimum Essential Medium (MEM) supplemented with 10% fetal bovine serum (FBS), 1 mM sodium pyruvate, and 1× nonessential amino acids (Thermo Fisher Scientific). Twenty-four hours after transfection, cells were collected for biochemical and patch clamp experiments. GFP-positive cells were used for whole-cell patch clamp recordings.

### Cell treatment

To activate PKC, PMA (Sigma-Aldrich) at various concentrations in culture media was used in Kv1.5-HEK cells for various periods at 37 °C. In total, 10 μM Bisindolylmaleimide I (BIM-1, ab144264, Abcam), 200 nM Sotrastaurin (16726, Cayman Chemical), and 200 nM Staurosporine (81590, Cayman Chemical) were used to inhibit PKC. Cells were incubated with a proteasomal inhibitor MG132 (10 μM, Sigma-Aldrich) or a lysosomal inhibitor bafilomycin A1 (Baf, 1 μM, Sigma-Aldrich) in culture media with PMA to block the proteasome and lysosome.

### Neonatal rat ventricular myocyte isolation and transfection

Experiments using rats were approved by the Queen's University Animal Care Committee and conducted in conformity with the Canadian Council on Animal Care. Sprague-Dawley neonatal rats of either sex at 1 day of age were sacrificed by decapitation followed by heart removal. Ventricular myocytes were isolated by enzymatic dissociation as described in detail recently (60). Myocytes were cultured in 10% FBS-containing Dulbecco's modified Eagle's medium/Ham's F-12 medium (Invitrogen) on coverslips overnight. Twenty-four hours after cell isolation, neonatal rat ventricular cardiomyocytes were transfected with Kv1.5 plasmid using Lipofectamine 2000.

### Western blot analysis

Whole-cell lysates from cultured cells were used for western blot analysis (20). Cells were washed with ice-cold phosphate buffer solution (PBS), collected, and centrifuged at 100*g* for 4 min. Next, the cell pellets were lysed using high-frequency sonication in ice-cold lysis buffer containing 1% PMSF and 4% protease inhibitor cocktail. The lysates were centrifuged at 10,000*g* for 10 min, and supernatants containing proteins were collected. The protein concentrations were determined using a DC Protein Assay kit (Bio-Rad). Appropriate amounts of double-distilled water and loading buffer containing 5% β-mercaptoethanol were added to the protein to make 0.3 μg/μl samples. Protein samples of 15 μg were loaded and separated on SDS-polyacrylamide gels and transferred onto polyvinylidene difluoride (PVDF) membranes (Millipore). The BLUeye Prestained Protein Ladder was used to identify the mass of proteins. The membranes were blocked to prevent nonspecific protein interactions with 5% nonfat skim milk and 0.1% Tween-20 in Tris-buffered saline (TBST) for 1 h at room temperature. Next, membranes were incubated with appropriate primary antibodies for 1 h at room temperature. Actin was detected as a loading control. The membranes were then incubated with horseradish peroxidase (HRP)-conjugated secondary antibodies (Cell Signaling Technology) for 1 h at room temperature. An ECL (enhanced chemiluminescence) detection kit (GE Healthcare) was used to visualize the protein bands on X-ray films (Fujifilm).

### Electrophysiological recordings

Currents were recorded using whole-cell patch clamp method. Cells were settled on the bottom of a 0.5 ml perfusion chamber in the bath solution. Patch glass pipettes were pulled using thin-walled borosilicate glass (World Precision Instruments). The pipettes had inner diameters of 1.5 μm and resistances of 2 MΩ when filled with solution. An Axopatch 200B amplifier and pCLAMP10 (Molecular Devices) were used for data acquisition and analysis. Data were sampled at 20 kHz and filtered at 5 kHz. Series resistance (Rs) was compensated by 80%, and leak subtraction was not used. All currents were elicited from a holding potential of –80 mV by depolarizing steps to voltages between –70 and +70 mV in 10 mV increments for 200 ms. A repolarizing step to −30 mV was applied for 250 ms before returning to the holding potential. To construct the voltage–current (I-V) relationships, the maximal currents during depolarizing steps were plotted against depolarizing voltages in each cell and summarized in each of the groups. The bath solution contained 135 mM NaCl, 5 mM KCl, 1 mM MgCl_2_, 2 mM CaCl_2_, 10 mM glucose, and 10 mM HEPES (pH 7.4 with NaOH). The pipette solution consisted of 135 mM KCl, 5 mM MgATP, 5 mM EGTA, and 10 mM HEPES (pH 7.2 with KOH). Patch clamp experiments were performed at room temperature (22 ± 1 °C).

### Immunofluorescence microscopy

HEK cells expressing WT Kv1.5 were grown on glass coverslips, then were exposed to MEM with or without PMA under various conditions. For membrane staining, the cells were incubated with Texas Red X-conjugated wheat germ agglutinin (WGA, 2.5 μg/ml; Invitrogen) for 1 min at room temperature prior to cell fixation. Cells were washed with PBS and fixed using 4% ice-cold paraformaldehyde in PBS for 15 min, permeabilized with 0.1% Triton X-100 for 10 min, and blocked with 5% bovine serum albumin in PBS for 1 h. Kv1.5 was labeled with rabbit anti-Kv1.5 primary antibody (H-120, sc-25681, Santa Cruz Biotechnology Inc) and Alexa Fluor 488-conjugated donkey anti-rabbit secondary antibody (A21206, Invitrogen). The coverslips were mounted onto glass slides using Prolong Gold Antifade reagent. Images were acquired using a Quorum Wave Effects Spinning Disc Confocal microscope at Queen's University Cancer Research Institute Image Centre.

### siRNA transfection

Endogenous Vps24 in Kv1.5-HEK cells was knocked down using the Vps24 siRNA (Sigma). Scrambled siRNA was used as the control. Cells were grown in 60-mm dishes at 60∼70% confluence and 100 pmoL of siRNA was transfected into cells using Lipofectamine 2000 (Invitrogen). Forty-eight hours after transfection, cells were subsequently cultured in MEM with 10 nM PMA for 3 h and were harvested for further experiments.

### Quantitative real-time polymerase chain reaction (qPCR)

Total RNA was extracted from Kv1.5-HEK cells using a Total RNA Mini Kit (Geneaid Biotech Ltd). RNA concentration was measured using an ND-1000 Spectrophotometer (NanoDrop Technologies). After treatment with DNase I (M0303S, New England Biolabs), the Omniscript Reverse Transcription Kit (Qiagen) was used for reverse transcription of total RNA (1 μg) to cDNA. Quantitative real-time PCR was performed with SensiFAST SYBR Hi-ROX Kit (Bioline Reagents, Ltd) and a Model 7500 thermal cycler (Applied Biosystems). For the internal control, a housekeeping gene *β-actin* was used. The primers used for *KCNA5* gene including forward sequence (5′ to 3′): GTTCCGCATCTTCAAGCTCTCC, and reverse sequence (5′ to 3′): CGAAGTAGACGGCACTGGAGAA; for *β-actin* gene including forward sequence (5′ to 3′): TGGCACCCAGCACAATGAA, and reverse sequence (5′ to 3′): CTAAGTCATAGTCCGCCTAGAAGCA. The real-time PCR protocol was: 2 min at 50 °C, then 10 min at 95 °C, followed by 40 cycles of denaturation at 95 °C for 15 s and annealing/extension at 60 °C for 1 min. Each PCR reaction was performed in duplicate. The cycle threshold (Ct) values of *β-actin* mRNA levels were not changed by PMA treatment. The transcription levels of *KCNA5* gene were calculated using the 2^−ΔΔCt^ method (61) using *β-actin* housekeeping gene. Data were expressed as fold changes of *KCNA5* gene normalized to the mean value of untreated controls.

### Recording I_Kv1.5_ in human iPSC-derived atrial cardiomyocytes

Human iPSC-derived atrial cardiomyocytes (ax2518) were obtained from Axol Bioscience (Little Chesterford) and grown on glass 18 mm circular coverslips following the manufacturer's instructions. Briefly, coverslips in 24-well plates were coated using 1× fibronectin coating solution (ax0049, Axol Bioscience) overnight at 37 °C in a humidified incubator. Cells were quickly thawed in a 37 °C water bath, then transferred to a sterile centrifuge tube, and diluted slowly with 9 ml plating medium (consisting of maintenance medium [ax2530, Axol Bioscience] with 10 μM Y-27632 [Sigma-Aldrich] and 10% FBS [Thermo-Fisher Scientific]). Cells were centrifuged at 200*g* for 5 min, the supernatant was discarded, and the pellet was resuspended in 1 ml plating medium for cell count to determine density. Cells were seeded at 1000 cells/cm^2^ in plating medium onto the prepared coverslips in 24-well plates after removing excess fibronectin from the wells. Cells were cultured at 37 °C, 5% CO_2_. Twenty-four hours after initial plating, the plating medium was replaced with maintenance medium (ax2530, Axol Bioscience) to remove any dead cells; the final cell density was around 500 cells/cm^2^. The culture medium was replaced with fresh maintenance medium every 48 h. Electrophysiology recordings were performed from day 3 to day 9 of culture. Most cells from day 3 to day 6 displayed the K^+^ current with biophysical characteristics of I_Kv1.5_. I_Kv1.5_ recorded from day 7 to day 9 became small and was not used. I_Kv1.5_ in human iPSC-derived atrial cardiomyocytes was further confirmed through its pharmacological properties (*e.g.*, sensitivity to 4-AP-mediated block). 4-AP was applied after control currents were recorded. Cells treated with PMA (10 nM) for 3 h were matched to control cells each time for whole-cell voltage clamp recordings. The currents were recorded with the standard external and pipette solutions used in HEK293 cells described above at room temperature (22 ± 1 °C).

### Reagents and antibodies

Scrambled siRNA, primary antibodies against Vps24 (C6, sc-271501), N-terminal specific mouse anti-Kv1.5 antibody (A3, sc-377110), rabbit anti-Kv1.5 antibody (H-120, sc-25681), goat anti-mouse (sc-2005), and goat anti-rabbit (sc-2004) IgG-HRP secondary antibodies were purchased from Santa Cruz Biotechnology Inc. The rabbit C-terminal-specific anti-Kv1.5 antibody (APC-004) was purchased from Alomone Labs. The horse anti-mouse (7076S) and goat anti-rabbit (7074S) HRP-linked secondary antibodies were purchased from Cell Signaling Technology. MEM, FBS, trypsin, sodium pyruvate, minimal essential amino acids, hVps24 siRNA (ID: SASI_Hs01_00160243), Lipofectamine 2000, Opti-MEM, Hank's Balanced Salt Solution were purchased from Invitrogen. G418, PMSF, protease inhibitor cocktail, β-mercaptoethanol, Triton X-100, BSA, Prolong Gold Antifade mountant, monoclonal mouse anti-actin antibody (A4700), mouse anti-FLAG antibody (F3165), and all chemicals/electrolytes used were obtained from Sigma-Aldrich. The BLUeye Prestained Protein Ladder (GeneDirex) was purchased from FroggaBio. X-ray films were from Fujifilm. Paraformaldehyde was obtained from Alfa Aesar.

### Statistical analysis

All data are expressed as the mean ± the standard deviation (SD). For western blots, box plots superimposed with original data points are shown, in which the line in the box shows the median, the small square shows the mean, and the bars show SD. For electrophysiological data, current densities (pA/pF) are plotted *versus* each voltage and shown as mean + SD. For experiments with multiple groups, a one-way analysis of variance (ANOVA) with Tukey's post-hoc test was used. For experiments where multiple groups were being compared with control, a one-way ANOVA with Dunnett's post-hoc test was used. For experiments between two groups, a two-tailed paired or unpaired Student's *t*-test was used. A *p*-value ≤ 0.05 was considered statistically significant.

## Data availability

All the data are in the article.

## Conflict of interest

The authors declare that they have no conflicts of interest with the contents of this article.
